# Health, consciousness, and the evolution of subjects

**DOI:** 10.1007/s11229-022-03998-z

**Published:** 2022-12-19

**Authors:** Walter Veit

**Affiliations:** 1 Department of Philosophy, University of Bristol, Bristol, UK; 2 Department of Biology, University of Oxford, Oxford, UK; 3 Munich Center for Mathematical Philosophy, Ludwig Maximilian University of Munich, Munich, Germany

**Keywords:** Darwinism, Health, Organisms, Consciousness, Pathological complexity

## Abstract

The goal of this programmatic paper is to highlight a close connection between the core problem in the philosophy of medicine, i.e. the concept of health, and the core problem of the philosophy of mind, i.e. the concept of consciousness. I show when we look at these phenomena together, taking the evolutionary perspective of modern state-based behavioural and life-history theory used as the teleonomic tool to Darwinize the agent- and subject-side of organisms, we will be in a better position to make sense of them both as natural phenomena.

## Introduction

As the title of this programmatic paper suggests, the following pages were motivated by the idea that when viewed through a Darwinian lens, we will see that health and consciousness are two closely related natural phenomena. I will argue that the origin and function of consciousness lie in the capacity to help complex but vulnerable animals to deal with and evaluate the species-specific health challenges that arise from the Darwinian problem of choosing the most adaptive actions among a set of alternative actions, as well as the avoidance of pathological behaviour. I will also show that that a biological science of consciousness must begin by appreciating the distinction between healthy and pathological variations of consciousness. Furthermore, I will argue that a naturalist understanding of this ‘biological normativity’ requires the development of a Darwinian theory of the organism that will in turn allow us to make sense of organisms as active *agents* and *subjects*, including their subjective experience as an integral part of our biological understanding of what makes a bat a bat, a snake a snake, and a healthy bee a healthy bee.^1^ By looking at the core problems in both the philosophy of medicine and philosophy of mind through the lens of the philosophy of biology, the present article thus hopes to feed two birds with one scone and use the resources of each debate to make progress on the other.

### Article outline

This programmatic paper is structured as follows. In Sect. 2, I motivate the project of this paper by discussing what it means to naturalize the notions of health and consciousness. In Sect. 3, I draw on the history of the Darwinian revolution to highlight the role of health in evolutionary thinking, discuss the endogenization of behaviour within Darwinism, and draw a number of useful lessons for a similar extension of Darwinism to endogenize consciousness. In Sect. 4, I draw on state-based and behavioural life-history theory to offer a means for a naturalization of health and consciousness within what I shall argue is the best theory of the organism that modern biology has to offer. Finally, I conclude the discussion in Sect. 5.

## Health and consciousness

One immediate philosophical problem for any biological investigation of consciousness and health is that the terms ‘consciousness’ and ‘health’ are notoriously ill-defined. The cognitive ethologist Frans de Waal (2016), for instance, notes that he prefers “not to make any firm statements about something as poorly defined as consciousness. No one seems to know what it is” (p. 23). Former zookeeper and animal welfare expert turned philosopher, Browning (2020), similarly expressed skepticism that health reflects “any naturally existing state”, instead of a mere cluster of different phenomena (p. 164). If they are right, the motivation of my article seems to rest on shaky ground; built to connect two natural phenomena that may not even exist.

But the absence of precise definitions for either should not stop us in our tracks. It is true that both terms, as used by the public, may be vague, ambiguous, and resistant to the analytical philosopher’s ideal of conceptual analysis. Indeed, if one’s goal is to provide definitions for these terms that would cover their varied usages, one may be tempted to conclude that we would be better off eliminating their folk concepts altogether.^2^

But my goal is not conceptual analysis, it is *conceptual explication* (Carnap, 1950); or as it has been called elsewhere, *naturalist conceptual engineering* (Veit & Browning, 2020). I am trying to capture a phenomenon in nature, for which the neurophilosopher Patricia Churchland (2002) suggests we should simply rely on common sense to establish “provisional agreement” on a number of “unproblematic examples of consciousness” (p. 133). There is no need to provide a philosophically satisfactory concept of consciousness or health before we can begin to investigate them, any more than we would need to define the concept of koala before we can learn about their enjoyment of eucalyptus leaves.

Scientists routinely proceed to investigate phenomena that have so far remained elusive, proving that vagueness need not be an obstacle to scientific inquiry (see Neto, 2020). In this naturalist activity, it is ultimately nature, not intuition, that will decide how we should understand consciousness, precisely because—as Figdor (2018) argues—we “lack widely accepted theories and models that can organize and articulate the pre-theoretic consciousness-related concepts we are using to guide our initial investigations” (p. 10). Following Churchland (2002), we can confidently reply that “we use the same strategy here as we use in the early stages of any science: delineate the paradigmatic cases, and then bootstrap our way up from there” (p. 133).

There are plenty of paradigmatic cases of consciousness: pains, pleasures, smells, visual experiences, tastes, a sense of one’s body, memories, alongside a whole other range of subjective experiences. Similarly, we have some intuitive grasp of instances of health and pathology in humans and animals alike, such as diseases, broken bones, lesions, parasites, burns, poisons, maladaptive behaviour, and other ‘biological wrongs’—even if we have struggled to derive something like a folk theory of health. So it is perhaps unsurprising that we can also intuitively distinguish healthy subjective experiences from unhealthy ones—such as major depressive disorder, anxiety disorder, aphantasia, synaesthesia, autism, schizophrenia, prosopagnosia, chronic pain, and many more. Yet, many philosophers of medicine would still outright *deny* that health is a purely natural phenomenon, which is why we should first look at this opposition a bit more closely, before I begin with my naturalization project.

### Resistance to naturalism in the philosophy of medicine

Despite the discussion of naturalist views by philosophers of medicine, their assessment is largely negative, and most within the field now maintain that health reflects personal evaluations or the values of society at large; a consensus that health is primarily a *normative* concept, rather than ‘only’ an objective *biological* property of organisms (Veit, 2021b, 2021c).^3^

Georges Canguilhem (1991), for instance, argued that “[t]here is no objective pathology. Structures or behaviors can be objectively described but they cannot be called [‘]pathological[’] on the strength of some purely objective criterion” (p. 226). Others like Lennart Nordenfelt (1995), who emphasize the concept of agency, have argued that health cannot be understood in a reductionist naturalist way and instead requires a more holistic conception, where it is understood as the ability to achieve one’s vital goals. Phenomenologists such as Carel (2007) have similarly argued that the “experience of illness cannot be captured within a naturalistic view” (p. 95). Such strong assertions against the very *possibility* of a naturalist account are surely premature and yet can be found throughout the literature, effectively making naturalism a ‘bogeyman’ of the field.

Rarely has there been a philosophical debate in which naturalism has been so unceremoniously dismissed, so it is perhaps not so surprising that philosophers haven’t sought to make sense of consciousness by linking it to health. Yet, this idea has been quite attractive among evolutionary biologists, such as Darwin’s protégé George Romanes, who speculated in some detail that pleasure and pain as sensations corresponding to the biological values of alternative actions may be the key to understanding the place of consciousness in nature:

Possibly, however—and as a mere matter of speculation, the possibility is worth stating—in whatever way the inconceivable connection between Body and Mind came to be established, the primary cause of its establishment, or of the *dawn of subjectivity*, may have been this very need of inducing organisms to avoid the deleterious, and to seek the beneficial; the raison d’être of Consciousness may have been that of supplying the condition to the *feeling of Pleasure and Pain*.

—George John Romanes (1883, p. 111) [italics added for emphasis].

Evolutionary thinking naturally lends itself towards a view in which the mind is born as a ‘device’ to track objective goods and biological wrongs and represent them in terms of hedonic valence (i.e. good, neutral, or bad feelings)—or as it has been called elsewhere: the evolution of sentient *Benthamite creatures* (Veit, 2022b). It does not appear at all mysterious from a Darwinian point of view that organisms would evolve to value states and behaviours that increase their own fitness and avoid those that are detrimental to their health. So why is there such a deep opposition to the view that health is a natural phenomenon?

The anti-naturalist consensus in the philosophy of medicine^4^ can be usefully summarized as an appeal to the ‘irreducibility’ of (i) the normativity of health and disease, (ii) the loss of agency in health and disease, and (iii) the phenomenology or subjective experience of health and disease. But who is to deny that these features can also be part of a naturalist account of health and disease? Naturalist philosophers have long worked on attempts to make these notions of normativity, agency, and phenomenal experience safe for naturalism. Notably, what all of these anti-naturalists share is an emphasis on *subjectivity*. Similarly to those who view naturalist explanations of consciousness as deeply problematic, they argue that the very idea of a purely naturalist account of health and disease is mistaken. They hold that one cannot account for health and disease from the objective third-person perspective of science, since they are phenomena at the level of a *subject*, not an *object*, and science cannot account for the former—a view familiar from so-called ‘naysayers’ who assert the impossibility of a scientific account of consciousness.^5^

This way of thinking about naturalism, however, is highly problematic regardless of whether it takes place within the philosophy of mind or the philosophy of medicine. Subjects aren’t some mysterious entities inaccessible to science: they are an evolutionary product and as well as the more familiar case of humans, also include many non-human animals (Godfrey-Smith, 2017). But the possibility of a Darwinian reconciliation between a view of health as a property of the organism as an ‘object’ and of the organism as a ‘subject’ has been given scant attention, precisely because *non-human health* has been less than an afterthought in this debate (see Matthewson & Griffiths, 2017).

As this article aims to show, the notions of health, pathology, and ‘biological wrong-goings’ are not only perfectly naturalistic concepts, but they play a key role in evolutionary biology, and will help us to extend the Darwinian revolution to include consciousness.

## The role of health in the Darwinian revolution

Whereas philosophers of medicine have been skeptical of naturalistic concepts of health, it is perhaps unsurprising that evolutionary biologists happily talk of health, pathology, diseases, damage, and other evaluative terms meant to refer to a perfectly naturalistic or purely biological sense of normativity. If there is natural design, then of course there are ways organisms ought to be and ways that things can go wrong for them. The endorsement of such a ‘teleonomic’ view of life is perhaps at the core of Darwinian thought, and yet it is often poorly understood or naively dismissed as ‘adaptationist’. Let us thus look directly examine the Darwinian paradigm.

### Darwinism, teleonomy, and natural design

In trying to view health and consciousness through a Darwinian lens we have to clarify what we mean by such a project, especially since I see the Darwinian approach in this paper as providing a ‘teleonomic’ alternative to a false dilemma between ‘externalist’ and ‘internalist’ approaches to consciousness. Some clarifications of these terms are in order before I proceed.

By ‘teleonomic’ I am employing Pittendrigh’s (1958) coinage of the term, as a naturalistically unproblematic Darwinian replacement for older and mistaken teleological notions about the purposefulness, design, adaptation, and normativity of life through recourse to evolutionary theory. Further, ‘internalist’ explanations can be understood as seeking to explain features of a system in virtue of other features of that system—of processes, structures, organization, and development *within* it, rather than outside of it. ‘Externalist’ explanations, on the other hand, aim to explain features of the system by recourse to the external, i.e. the environment—Godfrey-Smith (2002) calls them ‘outside-in’ explanations (p. 30). This distinction is not only relevant for categorizing different views of the mind, but also of life itself, since many (mistakenly) treat Darwinism as an externalist program, which I suspect is part of the reason why there is a lot of opposition to evolutionary approaches to health and consciousness.

Richard Lewontin has perhaps stated the alleged link between Darwinism and externalism the most forcefully, arguing that the success of the Darwinian project was due to its disentangling of the internal and external forces that had previously been seen as inseparable.^6^ Darwin broke with what Lewontin called *transformational* theories of the past, such as Lamarck’s (1984) theory of evolution that postulated change to individuals within their life-histories arising from ‘subjective’ (or what we may want to call ‘internal’) forces, such as will and striving. The Darwinian theory of the organism made it the “*object*, not the subject, of evolutionary forces” such as natural selection and random drift that are “autonomous and alienated from the organism as a whole” (Lewontin, 1985, p. 85). To complete the Darwinian revolution, however, Lewontin maintained that the internal forces—the subject-side of organisms—must be re-introduced:

Darwinism cannot be carried to completion unless the organism is reintegrated with the inner and outer forces, of which it is both the subject and the object.

—Richard C. Lewontin in (Levins & Lewontin, 1985, p. 106).

By this, Lewontin admittedly did not mean subjective experience, but rather how organisms as agents actively ‘participate’ in their evolutionary path and ‘construct’ their environments, as an alternative to a traditional adaptationist view of life. These notions of agency and construction have been highly influential in modern attacks on Darwinism (Ho & Saunders, 1979; Laland et al., 2014; Noble, 2015; Müller, 2017), but I am not here interested in the conceptual role of organisms as subjects for challenging the theoretical modeling of evolution. My interest lies in subjects as an evolutionary product, to allow us to make sense of the evolution of subjective experience. As Godfrey-Smith (2017) notes in his discussion of Lewontin’s earlier paper, subjects are not only a cause of evolutionary change, they are also its product.

In advancing a gradualist view of the evolution of consciousness, we can follow Godfrey-Smith’s suggestion to employ theoretically less loaded terms like ‘agency’ and ‘subjectivity’ as useful tools for thinking about organisms as being more or less subject-like: they “can realize subjectivity to a greater or lesser degree” (2017, p. 1). While subjectivity may appear as elusive as consciousness, it does not suffer similarly from an overabundance of theoretical frameworks. We can, as Godfrey-Smith (2019) notes, use it to bridge the gap between matter and mind: “[t]he history of life includes the history of subjectivity, and subjective experience is the experience *of a subject*” (p. 2). And in doing so we may be able to truly carry the Darwinian revolution to its completion.

Unlike Lewontin, however, I do not see a conflict between adaptationism and an explication of the subject-side of organisms. As this paper hopes to demonstrate, it is precisely with a Darwinian view of organisms that we will be able to make sense of ‘subjectivity’. This does not mean that we can’t recognize that evolutionary biology has been dominated by externalist modes of explanation, with features of the organism being explained in terms of their adaptive fit to their external environment.^7^ Evolutionary biologists readily admit that “[t]he suspicion of internal causes in the dominant neo-Darwinian culture ran so deep that every internalist idea, no matter how reasonable, was treated as an appeal to vitalism” (Stoltzfus, 2019, p. 46). But we should distinguish the idealization choices made by modellers from a deeper commitment to the necessity of an externalist view of adaptations. Indeed, it is a mistake to think of adaptationism and externalism as a onepackage-deal. We can straightforwardly follow Sterelny’s (1997) suggestion to decouple adaptationism from externalism and consider the two separately.

Many of the arguments against adaptationism are really arguments against its externalist versions, those that use a so-called ‘lock and key’ model of the adaptation between organism and environment; a criticism that need not apply to other versions. Modern evolutionary biology recognizes plenty of feedback between organisms and the species-specific environments in which natural selection takes place, such as Brandon’s (1990) notions of ‘selective environments’ and ‘ecological environments’, which can be distinguished from an organism-neutral externalist view of the environment. The external features that *matter* to the evolutionary trajectory of the organism are themselves causally dependent on the organism. No longer do modern evolutionary biologists see adaptations in the externalist design-sense such as that of the natural theologist William Paley, who argued for the presence of telos in nature and in turn used this as an argument for the existence of God as the designer.

As with many scientific concepts, the concept of adaptation came to be redefined—or rather explicated—in a naturalistically unproblematic sense, referring to whatever is produced by natural selection, even if such ‘design’ appears inefficient and wasteful (Griffiths & Gray, 2001, p. 209). Much of the opposition from ‘Neo-Darwinians’ to Gould and Lewontin’s (1979) criticism of ‘adaptation’ was based on a mismatch between a usage of that term in its original pre-Darwinian sense and its modern explication, which already included at least some of the features of feedback between organism and environment that were alleged to be lacking in the modern Neo-Darwinian view of life. We should not see Darwinism as an externalist theory of organismal traits that replaced the previous vitalist and romanticist modes of thinking that were confused between internal and external forces. Instead, we should see it as a *teleonomic* rejection of a false dilemma between internalist theories such as Lamarck’s and a strongly externalist view of organisms having been designed by a benevolent God to fit their environments. It achieves this through providing us with an inherently ‘dynamic’ or ‘interactionist’ picture of the living world, as is also emphasized in the field of evolutionary medicine (Nesse & Williams, 1998).

By understanding organisms as goal-directed systems or Darwinian *agents* evolved to maximize their fitness, our understanding of health, just like our understanding of adaptation and design, will come to be transformed. As I shall shortly demonstrate here, we can build a theory of the organism as both an object and *subject* with the tools of modern state-based and behavioural life-history theory, which does not—as Lewontin objected to—treat organisms as machines with mosaic-like traits, but rather as agents having to deal with integrated bundles of trade-offs in organismal design. As I shall argue, it is precisely this teleonomic theory that will bring out the subject-side of organisms.

Furthermore, as I shall show in the next section, the work of the classical ethologists nicely demonstrates how the Darwinian revolution only came to be extended towards behaviour through an appreciation of the distinction between healthy and pathological variations.

### Ethology, health, and the Darwinization of behaviour

Sterelny and Griffiths (1999) once described ethology as “the study of animal behavior under its normal ecological conditions (as opposed to unusual laboratory conditions) and from an evolutionary perspective” (p. 385). And this is certainly how many now think about it, as a tradition that stood in opposition to the lack of ecological and evolutionary thinking shown by the behaviourists, and one that has now largely been superseded by behavioural ecology. But there was a more philosophical conviction that motivated its founders, one of a teleonomic view of life, and this has largely gone unnoticed.

Both the ethologists and the behaviourists wanted to establish an objective science of behaviour in which we rely on a bottom-up approach that emphasizes the study of simple behaviours in order to understand more complex ones. But the ethologists hardly saw the behaviourists as Darwinians at all. This is ironic, considering that both the (early) behaviourists and ethologists used Darwin to motivate their approach. However, we can readily resolve this puzzle. Whereas the behaviourists emphasized the alleged externalist explanatory style of Darwin’s theory of natural selection, ethologists emphasized the theory itself, with its emphasis on function, survival value, and evolutionary phylogeny as sources of mechanisms to deal with the environments faced by organisms. This teleonomic perspective is nicely drawn out in a press release from the Karolinska Institute, which described their approach as a Darwinian way out of a dilemma between the behaviourist’s externalism and the vitalist’s insistence on internalist forces:

During the first decades of this century research concerning animal behaviour was on its way to be stuck in a blind alley. The vitalists believed in the instincts as mystical, wise and inexplicable forces inherent in the organism, governing the behaviour of the individual. On the other hand reflexologists interpreted behaviour in an one-side mechanical way, and behaviourists were preoccupied with learning as an explanation of all behavioural variations. The way out of this dilemma was indicated by investigators who focused on the *survival value* of various behaviour patterns in their studies of species differences. Behaviour patterns become explicable when interpreted as the result of *natural selection*, analogous with anatomical and physiological characteristics.

—Nobel Prize Outreach (2021) [italics added for emphasis].

Following the end of World War I, many psychologists - especially in America—rejected the previously widely accepted idea of human instinct (Griffiths, 2008). Instincts—in the sense of *unlearned* responses—came to be viewed with unease, due to their teleological and internalist nature and their inability to yield themselves to physiological investigation (and thus likewise, causal explanation), in addition to their being tied up with mistaken purposive and vitalist conceptions of life (Dunlap, 1919; Kuo, 1921; Tolman, 1923; Griffiths, 2008). Whereas the likes of Darwin strongly endorsed the idea of instincts, with the externalist turn of the behaviourists the notion came to be seen as unscientific. Lorenz, however, resisted this response as something that went too far in the opposite direction, maintaining that “it is hardly an exaggeration to say that the large and immeasurably fertile field which innate behaviour offers to analytic research was left unploughed because it lay, as no man’s land, between the two fronts of the antagonistic opinions of vitalists and mechanists” (1950, p. 232). Indeed, Lorenz simply tried to do for behaviour what Darwin’s naturalism had previously achieved for a similar false dilemma between vitalists and mechanists on the nature of life and organismic activity, i.e. to emphasize the teleonomic nature of animals:

[B]oth mechanists and vitalists were incurably inhibited by quite specific conceptual errors and prejudices that were magnified by their clash of opinion. This prevented them from initiating research into animal and human behavior at the point where it should have begun, namely with straightforward, unprejudiced *observation* of healthy animals living under normal conditions. They were quite incapable of seeing behavior for what it is, that is, as an extremely complex, organic *systemic entity* consisting of quite different components; one which, like *any* organic system, owes its particular constitution to a quite specific historical process of development.

—Konrad Lorenz (1997, p. 213) [italics in original].

To understand ethology as the mere opposition to work performed exclusively in the laboratory would be to miss out on this most important observation: ethology was intended as a teleonomic science (see also Thompson, 1986a, 1986b). In claiming to study learning in healthy organisms, the behaviourists did not recognize that it made no sense to think of health outside of the ecological context that animals evolved in. As Lorenz (1981) put it: “the pathologic can be defined only by having recourse to ecological concepts” (Lorenz, 1981, p. 57).

Unfortunately, this philosophical insight of the ethological tradition has come to be neglected, next to the more popular methodological slogan of studying animals in the wild, despite the fact that it was precisely their teleonomic reasoning that made them emphasize the importance of studying the lifestyles of healthy animals in their natural environments. This is why Lorenz praised Oskar Heinroth as the real founder of ethology as the comparative study of behaviour:

Accordingly, a finely developed sensitivity to the delicate and *often diffuse* boundary between the not-quite-normal and the already pathological is perhaps the most important talent that a scientific animal keeper must possess! On the other hand, however, the keeping of animals in our sense is an apprenticeship that renders one’s feel for the pathological just as acute as actual training in a medical clinic. It is surely no coincidence that Heinroth, the outstanding master of animal keeping as a scientific method, was, like the writer of this text, initially trained as a *medical doctor*.

—Konrad Lorenz (1997, p. 229) [italics in original].

The combination of observation and adaptationist thinking was inspired by the descriptive natural history activity of Darwin and the taxonomic activities that made a distinction between healthy and pathological specimens in order to describe what a ‘normal’ individual ought to look like. This was how the Darwinian revolution began, and this was thus how Lorenz thought a biological study of behaviour must get off the ground. It must be able to distinguish healthy from pathological behaviour, just as physiology and taxonomy have distinguished healthy from unhealthy phenotypes. This is why he emphasized early on that behaviour could be treated in just the same way as any other adaptive phenotype:

What behaviorists exclude from the narrow circle of their interest is not only other learning processes, but simply everything that is not contained in the process of learning by reinforcement-and this neglected remainder is *neither more nor less than the whole of the remaining organism*! […] What remains uninvestigated is all that makes an octopus an octopus, a pigeon a pigeon, a rat a rat, or a man a man, and, most important of all, what makes a healthy man a healthy man, and an unhealthy man a patient.

—Konrad Lorenz (1981, p. 71) [italics added for emphasis]

This is the basic motivation behind the ethologists’ Darwinization of behaviour and it is unfortunately a lesson the science of consciousness has not yet learned. If we are interested in making progress on the problems of consciousness within the next century, we must follow the ethologists’ dictum to distinguish healthy from pathological variations of consciousness by thinking about their survival value in an ecological context.

### Carrying Darwinism to completion

Throughout this section, my goal has been to emphasize the need for a naturalist approach to the place of consciousness in nature. By this I do not (only) mean the now common understanding of naturalistic thinking as the need for a strong continuity between science and philosophy, but rather the older meaning, of a *natural history* approach that begins with the careful observation of the diversity of organisms in the wild. It is in this intellectual tradition of taxonomic classifications and the mapping out of the life-histories of different organisms, rather than in laboratory experiments, that the Darwinian revolution began and changed biology forever. In order to understand organisms, the natural historians began by meticulously describing the living world. This led them to appreciate the distinction between healthy and pathological variations, and eventually to a teleonomic theory to make sense of this biological normativity in a naturalistically unproblematic manner.

Hence, it ought not to be all that surprising that the natural historian Alfred Russell Wallace came up with the idea of evolution by natural selection independently from Darwin, while suffering from a fit of malaria fever on his explorations (Meyer, 1895). Convinced of the common origin of species, but lacking a process explanation, the fragility of his own health and vigour made him realize that varieties within populations would lead to differences in the adaptive fit of organisms to their environments and thus change species in a “struggle for existence” (Darwin & Wallace, 1858, p. 54).^8^ Furthermore, just as Heinroth began his studies in medicine—and was praised by Lorenz for using his understanding of the difficult boundary between the normal and the pathological to advance the Darwinian revolution towards behaviour through his careful study of the life-history of birds—Darwin himself pursued a degree in medicine. While Darwin even grew up in a family of physicians, he eventually gave up on the pursuit of medicine, due to his distaste for operations and his greater obsession for natural history (Antolin, 2011). Nevertheless, it is hard to believe that an appreciation for the healthy and pathological varieties of organisms did not leave an impact on his teleonomic thinking about the appearance of ‘design’ in nature. After all, Darwin (1859) himself maintained that we can comfort ourselves that despite the great destruction in the struggle for life, “the vigorous, the healthy, and the happy survive and multiply” (p. 29). Unfortunately, very little attention has been given to the deep link between health, evolutionary theory, and natural history outside of the evolutionary medicine movement and parts of ethology.^9^

In order to understand healthy organisms in their natural environments, ethologists explicated the life-histories of organisms through observation and the creation of so-called ‘ethograms’, which were intended as an objective description of an organism’s behavioral repertoire during their lifetime. Just as the taxonomists and natural historians prior to Darwin distinguished normal from pathological organisms, ethologists aimed to begin a Darwinian study of behaviour by understanding natural behaviour. It will therefore hardly be surprising that ethograms are still a common tool for both veterinarians and animal welfare scientists for detecting abnormal or pathological behaviour in animals, such as tail-biting (Brunberg et al., 2011) and feather pecking (Sherwin et al., 2010). Yet, this is not a theory of health; ethograms only constitute a list of healthy and pathological behaviours.

It may thus not be surprising that many practitioners such as medical professionals, veterinarians, and animal welfare specialists such as Browning confidently treat health as something like a mere construct rather than a genuine integrated phenomenon in nature. They grant that we can measure parasite load, lack of nutrients, cancer, and presence particular diseases, among other vulnerabilities and dysfunctions, and that we could rank an animal’s ‘health’ with respect to each, but they would deny that there is some objective means of integrating them meaningfully into a single state. Something more is needed to naturalize health as genuine whole-organism phenomenon in nature and for this we unsurprisingly require a theory of the organism.

This insight has notably already been made by Darwin’s grandfather Erasmus Darwin, who was a very influential physician in a family of medical doctors and who shared an acute interest in natural history. At this time, natural history was often studied as a resource for medicine and he even anticipated some (unironically) ‘proto-Darwinian’ ideas in his natural history treatise *Zoonomia* that set out “to reduce the facts belonging to ANIMAL LIFE into classes, orders, genera, and species; and by comparing them with each other, to unravel the theory of diseases” (Darwin, 1794, p. 1).^10^ Such a theory was ultimately derived by Darwin, achieving his grandfather’s goal of providing a “theory founded upon nature, that should bind together the scattered facts of medical knowledge, and converge into one point of view the laws of organic life” (Darwin, 1794, p. 1).^11^

While Darwin himself had little to say about health and pathology, his teleonomic theory of evolution by natural selection was later used by the ethologists to likewise synthesize our scattered knowledge about normal and pathological behaviour into the very same evolutionary framework, casting their Nobel Prize for Physiology *or* Medicine in a particularly interesting light. Our folk understanding of ‘health’ as a biological phenomenon is revised in the light of evolutionary theory, just like that of ‘design’. To advance the goal of a true biological science of consciousness, we must similarly to the ethologists—make use of Darwin’s theoretical framework to synthesize our scattered knowledge about healthy and pathological cases of phenomenological complexity across the tree of life. This will allow us to integrate consciousness into the Darwinian revolution and complete our understanding of organisms such as electrosensing platypuses, echolocating bats, and infrared-sensing snakes. Or to borrow the words of Lewontin: in order to carry Darwinism to completion we must understand the organism as both an object and a subject.

Since I’ve repeatedly stated that state-based behavioural and life-history theory is the key theoretical resource for this task, as the theory of teleonomic agency, I will now move on to explain this theory in more detail and connect it to the foregoing lessons about the Darwinian revolution.

## A state-based behavioural and life-history theory of the organism

The twenty-first century equivalent of the ethologists’ attempt at building ethograms of organisms is modern state-based behavioural and life-history theory. All organisms go through a life cycle in which they are born, take in nutrients, grow, reproduce (whether sexually or asexually), and ultimately die (if death doesn’t occur before the completion of their life cycle). The diversity of life is essentially a diversity of different life-history strategies, which life-history theory aims to explain, thus making it “*the* integrative concept of organismic biology” (Kappeler, 2021, p. 34). In the design-space of organisms, there is a seemingly limitless room for the combinations of different traits that will lend themselves to different species-specific life-history strategies as optimal design-solutions to the challenges they are faced with.

Unfortunately, philosophers of biology, with the exception of Griffiths (and his recent collaborators), have given very little attention to this theory. Inspired by a (2018) talk of his that urged us to use life-history theory as a theory of the organism as a goal-directed system to solve the problem of distinguishing healthy from pathological traits, I aim to make progress here on the goal of developing a teleonomic theory of the organism as both an object and subject. This will also provide us with an account of health as a natural phenomenon by drawing on a modern extension of this theory: state-based behavioural and life-history theory.

### Life-history theory, agency, and adaptationism

Originally, life-history theory was largely concerned with fairly simple models (both discrete and continuous) of the simultaneous optimization of the survival-probability at different life-stages and the number of offspring produced in each in order to maximize fitness across a lifetime (Stearns, 1992; Roff, 1992; Morbeck et al., 1997; Roff, 2002). State-dependent or state-based behavioural and life-history theory (Mangel & Clark, 1986; McNamara & Houston, 1996) is an important extension of this theoretical framework, since it can then be used to make health and biological normativity naturalistically unproblematic notions by paying attention to the states of organisms and their environments in formalizing their life history tradeoffs in the optimization of fitness. To understand an organism’s teleonomic design is to understand their species-specific trade-offs between costly investments of resources into development, fecundity, and survival, with fitness providing an ultimate ‘common currency’ for this economic decision-problem, or ‘game’ against nature. Hence, a full understanding of an organism would be an understanding of their life-history *strategy*. As McNamara and Houston (1996) nicely put it, life-history theory is a theory “concerned with strategic decisions over an organism’s lifetime” (p. 215). It involves a naturalistically unproblematic kind of goal-directedness, by treating the organism as an agent whose traits have been shaped by natural selection to contribute to the single goal of fitness-maximization:

In life-history theory, […] numerous aspects of an organism’s life-cycle, such as the timing of reproduction or the length of its immature phase, can be understood by treating the organism as if it were an agent trying to maximize its expected number of offspring-or some other appropriate fitness measure-and had devised a strategy for achieving that goal.

—Samir Okasha (2018, p. 10).

While adaptationism has often been criticized for atomistic thinking, life-history theory is essentially a ‘holistic’ kind of adaptationism in which organisms are not treated as a single adult phenotype, nor a mere robot-like bundle of traits as in Lewontin’s criticism of the ‘adaptationists’, but a functionally complex and vulnerable life-cycle—a *dynamic process* that is faced with trade-offs from birth to death, for which complex optimality problems have to be solved. In short: a life-history.

It is within such a theory that we can bring out the subject-side of organisms and satisfy Lewontin’s demand to bring Darwinism to completion by paying attention to the “functional needs” of the organism (1985, p. 85). Notwithstanding that adaptationist thinking *can* at times be misleading,^12^this teleonomic theory of organismal agency provides the ideal theoretical framework in which to think about the evolution of subjectivity.

### A teleonomic theory of organisms

It is perhaps surprising that philosophers of biology have given very little attention to this theoretical framework, since it is here that we are provided with a teleonomic - or for that matter, adaptationist—theory of the organism that enables us to distinguish biological normativity and functioning from all the other causal processes operating in living systems. No philosopher has made clearer the need for such thinking than Millikan (2002), who maintained that to understand life we need a way to distinguish the normative, functional, goal-directed processes within an organism from all the other causal processes or mere ‘noise’:

Living chunks of matter do not come, just as such, with instructions about what are allowable conditions of operation and what is to count as allowable input. Similarly, they do not come with instructions telling which changes to count as state changes within the system and which instead as damage, breakdowns or weardowns. Nor do they come with instructions about which processes either within the organism or outside it are to count as occurring within and which are irrelevant or accidental to the system.

—Ruth Garrett Millikan (2002, p. 121).

To distinguish what matters to an organism, what is pathological, and what is part of it, one must attend to the organism’s design and to the selective pressures faced by the organism. It is only within such a teleonomic theory of the organism that we can make sense of health and the subject-side of organisms. Physiologists may often succeed in progressing our understanding of the organism without evolutionary considerations, but once their work is intended to generalize they ought to recognize that the various processes of the organism must be understood in “the light of life history theory” (Griffiths, 2009, p. 23). Otherwise, we are inevitably led to make mistaken judgements about which variations are healthy and which are pathological.

Morbeck et al. (1997) elegantly describe life-history theory as providing us with “a means of addressing the integration of many layers of complexity of organisms and their worlds” (p. xi). This makes it the ideal agential framework to explicate the link between health and consciousness and make sense of the evolution of subjects and their experience. In thinking about the ecological lifestyles of different species it is thus not unreasonable to treat them as economic agents maximizing their utility (i.e. fitness). Each individual within a species is fundamentally faced with a resource allocation problem, though the solutions to this problem are admittedly more similar within a species than across species (Kappeler, 2021, p.35). This is the economy of nature.

It is no accident that ecological and economic models share many similarities and frequently borrow from each other. Under resource scarcity, there is a constant trade-off between the parameters of reproduction and survival: “reproduction in the current age class must be traded off against reproduction later; current reproduction must be also traded off against growth, and against condition (the maintenance of structures that have already developed); current growth or condition may trade off with survival to later age classes” (Griffiths & Matthewson, 2018, p. 319). Boorse (1977) once argued that one cannot build a theory of health from an evolutionary concept of adaptedness, since “parents hardly become healthier with each successive child, nor would anyone maintain that the healthiest traits are the ones that promote large families” (p. 548). But Griffiths and Matthewson (2018) are right to note that this is a very superficial take on evolution, insisting that there is a trade-off between offspring quality and offspring number, as the British evolutionary biologist David Lack^13^ demonstrated with his life-history work on optimal clutch size (p. 305).

It is unfortunate that philosophers have so far given scant attention to life-history theory, since it is within the context of this theory that many puzzling philosophical questions about the nature of organisms can be resolved, with fitness playing a crucial conceptual role for thinking about trade-offs in organismal design. By ignoring this framework—or for that matter, evolutionary biology at large—philosophers such as Boorse have made biologically uninformed statements about the viability of an evolutionary understanding of biological normativity. Let me thus now return to my promise of explicating the concept of health in a naturalistically unproblematic manner.

### Health and pathological complexity

Before we can think about what it means for an organism to be healthy, it is important to note that fitness is not just the number of offspring, despite reproduction being correctly identified as the ‘telos’ of the organism. Griffiths and Matthewson (2018) grant that Boorse’s comment may have been “light-hearted” but note that “comments such as this undoubtedly contributed to the premature rejection of evolutionary views of dysfunction” (p. 305). Indeed, they gave aid to a sphere in which a serious engagement with evolutionary theory could simply be waved away with a swift remark. But fitness is ultimately the common currency for making sense of how one organism can be healthier than another, when it comes to injuries, nutrient lack, or parasite load. A judgement over which organism is healthier than another must not restrict itself to only evaluating these dimensions individually. While the health of an organism is made of of a vast range of different components, they do form a naturally existing state that can be expressed in terms of life-history theory. The mere fact that health is made up out of multiple components is no reason to deny its existence as a organism-level phenomenon.

However, it is only by employing the Darwinian notion of biological fitness that we are provided with a common currency for what I call the ‘pathological complexity’ of different species, used to assess how badly (or well) things are going for an organism. It is the computational complexity of maximizing fitness given a particular life history strategy of a teleonomic living system, which can be mathematically expressed as the number of variables and constraints in the fitness-maximization problem of any given life-history strategy. So organisms within one and the same species can have a higher pathological complexity dependent on the presence of predators and the heterogeneity of the environment. Let me now address how we can use this notion to think about health.

My motivation here is that the biological world is too messy for simple binary categories, be that for consciousness or health, which is why I have proposed to naturalize them both in terms of phenomenological and pathological *complexity* (Veit, 2022a, 2022b, 2022f). An organism is not simply healthy or unhealthy, given that dysfunctions in complex teleonomic systems are unavoidable. Living systems are constantly faced with trade-offs: avoidance of one danger comes at the cost of exposing oneself to another, or entails foregoing some benefit. Health is simply a measure of how optimally an organism deals with the pathological complexity trade-offs it faces during its life cycle—or, to put it differently, how well an organism succeeds in its species-specific life-history strategy. I would like to point out, however, that I am employing the term ‘pathological complexity’ instead of the equally adequate terms ‘life-history complexity’ and ‘teleonomic complexity’ not because I want to say that organisms with more complex life history strategies are inherently less healthy, but because it is only in understanding the fundamental design trade-offs that life-history theory is meant to capture that we can distinguish pathological from healthy variations. This also goes for variations within consciousness, or as I prefer to call it ‘phenomenological complexity’ (Veit, 2022a).

As one of our reviewers noted, the way we usually think about health and fitness does not treat them as one and the same phenomenon. My response is twofold. Firstly, I am not here interested in how the concept of health is typically used in human discourse. What I am interested in is health as the natural phenomenon of biological design. Secondly, these notions are not intended to be the same, even if they are closely related in my picture. Fitness is the ultimate measure of biological success, but health is about how well an organism’s design achieves its life history strategy. All biological life is faced with design trade-offs and fitness provides a common currency for natural selection to ‘choose’ the best design. While this view of health may not fit the goals of many normativists in the philosophy of medicine, this is of no concern to my project here, which only aims to establish that there is a purely naturalist sense of health and biological normativity that plays an important role in understanding the living world.

Trade-offs of pathological complexity can occur on a genetic level, with selection pulling in different directions, and on a physiological level where—as Griffiths and Matthewson (2018) note—nutrients can be allocated to either somatic or germ cells. While these trade-offs can lead to health problems for organisms, my focus in this paper will be the organism as an integrated agent. Ernst Mayr (1988) once said that “the individual and not the gene must be considered the target of selection” (p. 101). Whether this is a general truth is legitimately contested, but in thinking about the evolution of conscious agency it is certainly the right approach. The reason organisms do not start to reproduce instantly from their birth onward, is a lack of resources. Analogous to the way in which a firm can be usefully treated as an agent, and must first gather resources, equipment, and manpower before selling on a market, organisms must first make an investment into growth before producing viable progeny.

Answering the question of how to solve this economic optimization problem under constraints, has been the original motivation for life-history theory. Griffiths and Matthewson (2018) point out that many organisms pursue a semelparous as opposed to a iteroparous strategy, i.e. “they complete all their growth before engaging in a single round of reproductive activity to which they commit all their resources” (p. 319). They mention Australian marsupials of the genus *Antechinus* as a core example for the death of males after a single breeding season, with one species even having all females dying after the weaning of their offspring.^14^ While “such behaviour in males may be seen as strikingly pathological, through life-history theory we can see that it is not. Their best response to their species-specific pathological complexity is to invest all their resources into reproduction in a single breeding season, and hence this not pathological” (Veit & Browning, 2022, p. 57). This example nicely illustrates how our folk understanding of health as a natural phenomenon of design ought to be updated in the light of evolution.

However, the *Antechinus* example is perhaps not the best illustration of an organism fine-tuned for a semelparous life-history, since the mammalian body-plan is relatively unsuited to this strategy—due both to the requirement of high investment into offspring, as well as an investment into an adaptive immune system, which is sometimes deliberately turned off during the breeding season, but lends itself to numerous reproductive cycles.^15^

Better examples are found in insects, where many species engage in such life-cycles because a semelparous strategy relies on relatively short lives, which is favoured by a high rate of juvenile survival but a high probability of death in adulthood, with bodies being discarded relatively quickly (Fritz et al., 1982). It is in this context that insects are often claimed to not feel pain, because they would not benefit from carrying such expensive equipment for their survival (see Godfrey-Smith, 2020). This could certainly be *one way* to respond to the pathological complexity of insect life, though I argue elsewhere against the notion that insects do not feel pleasure and pain (Veit, 2022c). It is true, however, that many insects play a so-called r-strategy as opposed to K-strategy, in which quantity as opposed to quality of offspring is maximized. In the extreme, there is only one reproductive season before the organism dies, sometimes also called ‘big bang reproducers’ (Diamond, 1982). Importantly, these should not be understood as binary distinctions, but rather as continuums along a single axis with two extremes. Furthermore, whereas many mammals stop growing after reaching a reproductive stage, many insects, fish, and reptiles continue to grow until death (Griffiths & Matthewson, 2018). These are only some among many other dimensions in which self-maintenance and reproduction can come into conflict, and it is only by taking an evolutionary perspective that we can understand health as a question of optimality of organismal design. Similar life-history trade-offs can be observed in plants, fungi, and even single-celled organisms, making pathological complexity a universal design problem that emerged at the very origins of life as a set of teleonomic problems under constraints that need to be solved simultaneously in order to maximize fitness.

### Pathological and phenomenological complexity

As I mentioned above, life-history models were originally fairly simple, assuming that age, reproduction, and self-maintenance can be modelled independently (i.e. that the individual differences between individuals at specific ages could be idealized away) (Pianka & Parker, 1975; McNamara & Houston, 1996). More recent work has expanded life-history theory to incorporate the behaviour, physiology, and environmental conditions of organisms, something that is now typically referred to as state-based behavioural and life-history theory (Mangel & Clark, 1986; McNamara & Houston, 1996). This gets us considerably closer in modeling pathological complexity as the fundamental problem of organismal trade-offs, but the problem also becomes computationally far more demanding.

To maximize fitness it is no longer just a problem of choosing a single strategy across a life-time, but also of choosing strategies at any moment of one’s life-cycle, depending on one’s bodily state and environmental conditions. The more degrees of freedom there are in the behavioural option space,^16^ the higher the pathological complexity of this fitness-maximization problem, since organisms have to make sure to make the right decisions at the right time, a choice that depends on their current state and that of the environment. Indeed, they are faced with a computational explosion of complexity. This complexity only increases when we add fluctuating environments such as changing weather conditions, food supply, risk of predation, and population density (e.g. Metz et al., 1992; McNamara, 1997; McNamara & Houston, 2008) and the frequency-dependence of optimal strategies, which has been extensively studied by evolutionary game theorists (Maynard Smith, 1987). These increases in pathological complexity become much harder to track for both the organism and the biologist, but that is not to say it isn’t there. Pathological complexity is the fundamental teleonomic challenge every organism has to deal with, and as I shall argue in a compendium article (Veit, 2022b), it is precisely due to an explosion of pathological complexity during the Cambrian that sentience became a worthwhile investment as an efficient capacity for consciously evaluating these continuous life-history trade-offs in action-selection.

As Romanes speculated, the origins of mind plausibly lie in enabling organisms to represent the biological normativity of their living situation to themselves: states are evaluated as good and bad and accordingly felt, with pleasure and pain providing something of a proximate common currency for animals to solve the trade-off problems arising from alternative actions. Furthermore, it is in this context that varieties of consciousness can be described as healthy and pathological, according to how well they enable an organism to succeed at its life-history strategy. This is precisely what we need in order to extend the Darwinian revolution towards consciousness and endogenize it within the explanatory scope of Darwinism. A more detailed examination of how this evolution of evaluative consciousness commenced is the subject of another paper of mine (Veit, 2022b). My goal here was only to highlight that it is by understanding health as a natural phenomenon, which requires us to develop a theory of the organism as both an object and subject, that we will be able to emulate the success of the ethologists and Darwinize subjective experience.

Lastly, those interested in the evolution of consciousness frequently talk of a special *lifestyle* or *mode of being*—an animal way of life emerging in the Cambrian that has given rise to minimal sentience (Ginsburg & Jablonka, 2019; Godfrey-Smith, 2020). Using life-history theory will help us to naturalize this idea in terms of the pathological complexity of this new lifestyle, and to assess the phenomenological complexity of animals around us here and now in terms of their distinct pathological complexity challenges - which I have done elsewhere in the case of insects and gastropods (Veit, 2022c). Indeed, what we can observe is that organisms with higher pathological complexity are inherently more subject-like, having to make complex decisions that address their conflicting functional needs. As I hope to have motivated in this programmatic paper, it is only within this evolutionary context of the optimal life-history strategies of biological agents, that we will understand organisms as objects as well as subjects, and be able to extend the Darwinian revolution towards consciousness. Thinking about their life history strategies and pathological complexity challenges will provide an excellent ecological and evolutionary lens to think about the possible adaptive benefits of different kinds of minds and thus move us towards a bottom-up Darwinian study of consciousness. While this kind of work may appear speculative at first, this is precisely how the Darwinian revolution began: by careful observation of the natural world and making a distinction between the healthy and pathological. And as I have shown in a series of publications applying the pathological complexity framework to think about the mind (Veit, 2022a, 2022b, 2022c, 2022f), a thorough ecological understanding of both human and non-human organisms will enable us to progress towards a true Darwinian science of the consciousness.

## Conclusion

This programmatic paper was inspired by both the Darwinian revolution and its extension towards behaviour, which both began with an appreciation of healthy and pathological variations in nature. By looking at the Darwinian revolution and its extension towards behaviour, I hope to have made clear that a similar approach is needed to extend the Darwinian revolution once again to endogenize consciousness within a Darwinian view of life.

In order to do this, I have argued that we should draw on state-based and behavioural life-history theory as the 21st century equivalent of the ethologist’s ethogram; a teleonomic theory of the organism that is able to help us naturalize the elusive normative and teleological properties of organisms as goal-directed systems. It is here that we find the best current biology to make these notions safe for naturalism and further move the philosophy of biology, along with the philosophy of medicine and of mind. In order to think about the mind from a teleonomic Darwinian perspective, life-history theory offers us the means for thinking about the role of the mind for the life history strategies of different organisms and the pathological complexity challenges they are faced with.

Many challenges lie ahead, but it is in this teleonomic theory of the organism that we will be able to make significant progress. In future work, including a book-length treatment (see Veit, 2022d, 2022e) will use this framework in collaboration with biologists, to develop a mathematical measurement of pathological complexity to assess the health of organisms and help us to think about the trade-offs inherent to biological design and behaviour. Additionally, the classification of varieties of mental traits into disorders of consciousness or psychiatric conditions more generally is one of the most challenging topics in the philosophy of medicine (and psychiatry). Using life-history theory will offer us an elegant teleonomic theory of organismal agency with which to apply the adaptationist lens of evolutionary theory, without suffering from the narrow externalist adaptationist thinking that has been prevalent in past applications of evolutionary thinking to the mind (see also Veit & Browning, forthcoming). It is here that we are provided with a theory that accounts for organisms as subjects as well as objects, thus bridging the gap between ‘objective’ and ‘subjective’ accounts of health and pathology.

If the mind evolved to keep track of the objective ‘goods’ and biological ‘wrongs’, there would be a straightforward link between sentience and biological normativity, providing a new domain in which a mismatch between the design of an organism and its goals may occur—further increasing pathological complexity—just as the evolution of behaviour did. Such a synthesis may force us to rethink older ways of understanding, but it is only in drawing on the best recent science that we can hope to defend a naturalist picture of the teleology, normativity, and the minds of living systems. Finally, I hope there is some truth in the synthesis of literatures I have offered here, and that it will inspire future work on the teleonomic nature of life and mind.

## Footnotes

1 I borrow this phrasing from an important quote by one of the ‘fathers of ethology’, Konrad Lorenz—one that will be discussed shortly.

2 Wilkes (1984), for instance, has argued so in the case of consciousness and Hesslow (1993) and Ereshefsky (2009) in the case of health.

3 See Dominic Murphy’s (2020) SEP article “Concepts of Disease and Health” for an excellent recent overview.

4 This is not to imply that there aren’t some philosophers who draw on evolution (e.g. Griffiths & Matthewson, 2018; Matthewson & Griffiths, 2017).

5 Flanagan (1991) offers an excellent critique of these consciousness skeptics.

6 See also Lewontin and Levins (1997).

7 See Williams (1966), Gould (1977), Gould and Lewontin (1979), Endler (1986), Godfrey-Smith (1996, 2001, 2002), Lewontin (1983), Mayr (1994), Walsh (2015), Chiu (2019).

8 In his biography of Wallace, Shermer (2002) offers an elegant recounting of how Wallace came to realize the phenomenon of natural selection (pp. 112–118).

9 Unfortunately, this also goes for evolutionary veterinary science (Veit & Browning, 2021).

10 Darwin denied that his grandfather’s work had a major influence on him, but there are many similarities between their views.

11 See also Nesse (2007).

12 For critical discussions of this kind of agential thinking common to evolutionary biology, see Okasha (2018), Veit (2021a, 2021b, 2021c).

13 See Lack (1947).

14 The Brown antechinus (*Antechinus stuartii*) being a paradigm example (see Dobson, 2013).

15 However, these animals provide an excellent case for how we could evaluate not only species- but also sex-dependent life-history strategies, including in humans: whereas female deviations from male ‘norms’ have historically been often labeled as ‘pathological’ in humans, an evolutionary understanding of their different pathological complexity challenges can reveal such differences to be adaptive and thus healthy (Veit & Browning, 2022).

16 To simplify a bit too much: how many alternative actions an organism can take.
